# Self-Assembling Imageable Silk Hydrogels for the Focal Treatment of Osteosarcoma

**DOI:** 10.3389/fcell.2022.698282

**Published:** 2022-06-20

**Authors:** Zhibin Peng, Ming Li, Yuan Wang, Hongbo Yang, Wei Wei, Min Liang, Jianhui Shi, Ruixuan Liu, Rui Li, Yubo Zhang, Jingsong Liu, Xu Shi, Ran Wan, Yao Fu, Rui Xie, Yansong Wang

**Affiliations:** ^1^ Department of Orthopedic Surgery, The First Affiliated Hospital of Harbin Medical University, Harbin Medical University, Harbin, China; ^2^ The Key Laboratory of Myocardial Ischemia, Ministry of Education, Harbin Medical University, Harbin, China; ^3^ Department of Critical Care Medicine, The Second Affiliated Hospital of Harbin Medical University, Harbin Medical University, Harbin, China; ^4^ Innovation and Entrepreneurship Square, Science and Technology Innovation City, Hi-Tech Zone, Harbin, China; ^5^ Department of Orthopedic Surgery, Affiliated Hospital of Chifeng University, Chifeng University, Chifeng, China; ^6^ Department of Orthopedic Surgery, Harbin 242 Hospital, Harbin, China; ^7^ Department of Orthopedic Surgery, Heilongjiang Provincial Hospital, Harbin, China; ^8^ Department of Digestive Internal Medicine and Photodynamic Therapy Center, Harbin Medical University Cancer Hospital, Harbin Medical University, Harbin, China

**Keywords:** iodine, minimally invasive, osteosarcoma, imageable hydrogel, silk fibroin

## Abstract

**Background:** The standard treatment for osteosarcoma comprises complete surgical resection and neoadjuvant chemotherapy, which may cause serious side effects and partial or total limb loss. Therefore, to avoid the disadvantages of traditional treatment, we developed self-assembling imageable silk hydrogels for osteosarcoma.

**Methods:** We analysed whether iodine induced apoptosis in MG-63 and Saos-2 cells by using CCK-8 and flow cytometry assays and transmission electron microscopy. Western blotting was used to analyse the pathway of iodine-induced apoptosis in osteosarcoma cells. PEG400, silk fibroin solution, polyvinylpyrrolidone iodine (PVP-I), and meglumine diatrizoate (MD) were mixed to produce an imageable hydrogel. A nude mouse model of osteosarcoma was established, and the hydrogel was injected locally into the interior of the osteosarcoma with X-ray guidance. The therapeutic effect and biosafety of the hydrogel were evaluated.

**Results:** Iodine treatment at 18 and 20 µM for 12 h resulted in cell survival rate reduced to 50 ± 2.1% and 50.5 ± 2.7% for MG-63 and Sao-2 cells, respectively (*p* < 0.01). The proportion of apoptotic cells was significantly higher in the iodine-treatment group than in the control group (*p* < 0.05), and apoptotic bodies were observed by transmission electron microscopy. Iodine could regulate the death receptor pathway and induce MG-63 and Saos-2 cell apoptosis. The hydrogels were simple to assemble, and gels could be formed within 38 min. A force of less than 50 N was required to inject the gels with a syringe. The hydrogels were readily loaded and led to sustained iodine release over 1 week. The osteosarcoma volume in the PEG-iodine-silk/MD hydrogel group was significantly smaller than that in the other three groups (*p* < 0.001). Caspase-3 and poly (ADP-ribose) polymerase (PARP) expression levels were significantly higher in the PEG-iodine-silk/MD hydrogel group than in the other three groups (*p* < 0.001). Haematoxylin and eosin (H&E) staining showed no abnormalities in the heart, liver, spleen, lung, kidney, pancreas or thyroid in any group.

**Conclusions:** Self-assembling imageable silk hydrogels could be injected locally into osteosarcoma tissues with X-ray assistance. With the advantages of good biosafety, low systemic toxicity and minimal invasiveness, self-assembling imageable silk hydrogels provide a promising approach for improving the locoregional control of osteosarcoma.

## Introduction

Osteosarcoma (OS), a malignant and highly metastatic cancer, is the most common type of primary malignant bone tumour in children and adolescents (Ottaviani and Jaffe, 2009; Arndt et al., 2012; Whelan et al., 2012; Cersosimo et al., 2020; Yang et al., 2020; Jiang et al., 2021). Conventional treatments for OS include neoadjuvant chemotherapy and a combination of complete surgical resection and adjuvant chemotherapy with drugs such as doxorubicin, cisplatin, methotrexate or ifosfamide (Muscolo, Ayerza, Aponte-Tinao, & Ranalletta, 2005; Anninga et al., 2011; Marec-Berard et al., 2020; Chen et al., 2021; Lin et al., 2021). However, chemotherapy often causes severe adverse systemic reactions and drug resistance. In addition, although limb salvage surgery is often performed, the resection surgery can still result in partial or total limb loss (Simon, Aschliman, Thomas, & Mankin, 1986; Rhodes, 1987; Bacci et al., 2002; Muscolo et al., 2005; Takeuchi et al., 2019). Therefore, the development of a new treatment for OS is critical.

Polyvinylpyrrolidone iodine (PVP-I) can effectively kill bacteria, viruses, fungi, and protozoa (Cunliffe and Fawcett, 2002; Lepelletier et al., 2020; Stathis et al., 2021). As a broad-spectrum antibacterial agent, it is routinely used to disinfect the skin and clean wounds. PVP-I can release free iodine, which has a strong antimicrobial effect. Similarly, free iodine is an effective tumoricidal agent and is becoming increasingly popular as an antitumour treatment in clinical practice. PVP-I has been widely used as an irrigation fluid during percutaneous endoscopic gastrostomy and in head, neck, and colorectal cancer surgery to eliminate free cancer cells to prevent implantation, metastasis and recurrence (Wu et al., 1998; Basha et al., 2000; Pattana-arun and Wolff, 2008; Hah et al., 2012; Chang et al., 2014). Furthermore, in a rat model study, researchers found that tumour formation can be impeded by the presence of iodine. These results indicate that iodine can prevent early cancer progression by exerting an inhibitory effect on cancer-initiating cells (Ghent et al., 1993; Funahashi et al., 1996; Kessler, 2004; Garcia-Solis et al., 2005). However, few studies have been conducted on the use of iodine as a treatment for OS, and the mechanism underlying the anti-OS effect of iodine is still unclear.

Cell death is divided into four types according to the appearance of the dead cells: type I cell death (apoptosis), type II cell death (autophagy), type III cell death (necrosis) and type IV cell death (pyroptosis) (Arndt et al., 2012). Apoptotic cell death is triggered by two main signalling pathways: the extrinsic pathway (the death receptor pathway) and the intrinsic pathway (the mitochondrial pathway) (Green and Llambi, 2015; Yuan et al., 2018). The death receptor pathway involves classical ligand-cell surface receptor interactions. Apoptotic cell death is mainly caused by either death receptors (DRs) or the mitochondrial pathway, although additional pathways exist. DRs, such as factor-associated suicide (Fas), cluster of differentiation 95 (CD95), trail receptor (TRAIL-R), and TNFR1, induce apoptosis by directly recruiting a caspase-activation platform upon binding to their respective ligand (Green and Llambi, 2015; Krishnan et al., 2019). The association of the receptor Fas with its ligand FasL triggers the DR pathway, which plays an important role in the immune regulation, development, and progression of cancers (Suda et al., 1993; Lee and Ferguson, 2003). Receptor clustering upon stimulation induces FADD and caspase-8 oligomerization, activating the caspase signalling pathway. Cleaved caspase-3 is a critical executioner of apoptosis, as it is either partially or totally responsible for the proteolytic cleavage of many key proteins, such as the nuclear enzyme Poly (ADP-ribose) polymerase (PARP) (Fernandes-Alnemri, Litwack, & Alnemri, 1994). PARP and cleaved PARP ultimately trigger apoptosis.

Meglumine diatrizoate (MD) is a water-soluble contrast agent, it is a colourless or faint yellow liquid. MD has a short half-life and is rapidly metabolized in the kidneys, and it has been widely used for imaging the digestive system, cardiovascular system, urinary system, and female reproductive system as well as for myelography but is mainly used for auxiliary diagnosis(White et al., 1993; Mercadante et al., 2004; Arndt et al., 2012; Khasawneh et al., 2013; Khaleel et al., 2019; Barret et al., 2020; Wu et al., 2020).

Silk fibroin is the major structural protein in silkworm cocoons. Due to their hypoimmunogenicity, biocompatibility and biodegradability, silk biomaterials have been widely used in tissue engineering and for drug delivery. A silk fibroin solution can form hydrogels after physical and chemical treatment, for example, treatment by sonication, vortexing, electrical fields, inorganic ions and macromolecular compounds (Wang et al., 2008; Yucel et al.,n, 2009; Leisk et al., 2010; Wang et al., 2015; Ma et al., 2016; Gholipourmalekabadi et al., 2020). Polyethylene glycol (PEG) is an organic compound that can be mixed with silk fibroin to form silk hydrogels. The gel formed by PEG and silk fibroin can be used as a sustained-release system to deliver biological factors and drugs (Seib et al., 2013; Wang et al., 2015; Ma et al., 2016; Turkkan et al., 2017; Boni et al., 2020). In addition, silk fibroin hydrogels can be delivered through syringes, allowing silk hydrogels to be delivered to the required site in a minimally invasive manner to deliver drugs or biological factors to the target area (Seib et al., 2013; Wang et al., 2015; Ma et al., 2016; Liang et al., 2019; Farokhi et al., 2020). Many researchers have used silk fibroin for sustained-release drug delivery to tumours. Silk fibroin was used in several forms in these studies, including nanoparticles (Kim et al., 2015; Yang et al., 2015; Li et al., 2016; Xie et al., 2017), films (Seib and Kaplan, 2012; Zhang et al., 2019), and hydrogels (Seib et al., 2013; Hong et al., 2020). Therefore, silk fibroin products (including hydrogels) can play an important role in inhibiting tumour growth by stably releasing drugs during tumour treatment. However, few studies have been conducted on the use of silk fibroin products for the treatment of OS.

**FIGURE 1:**
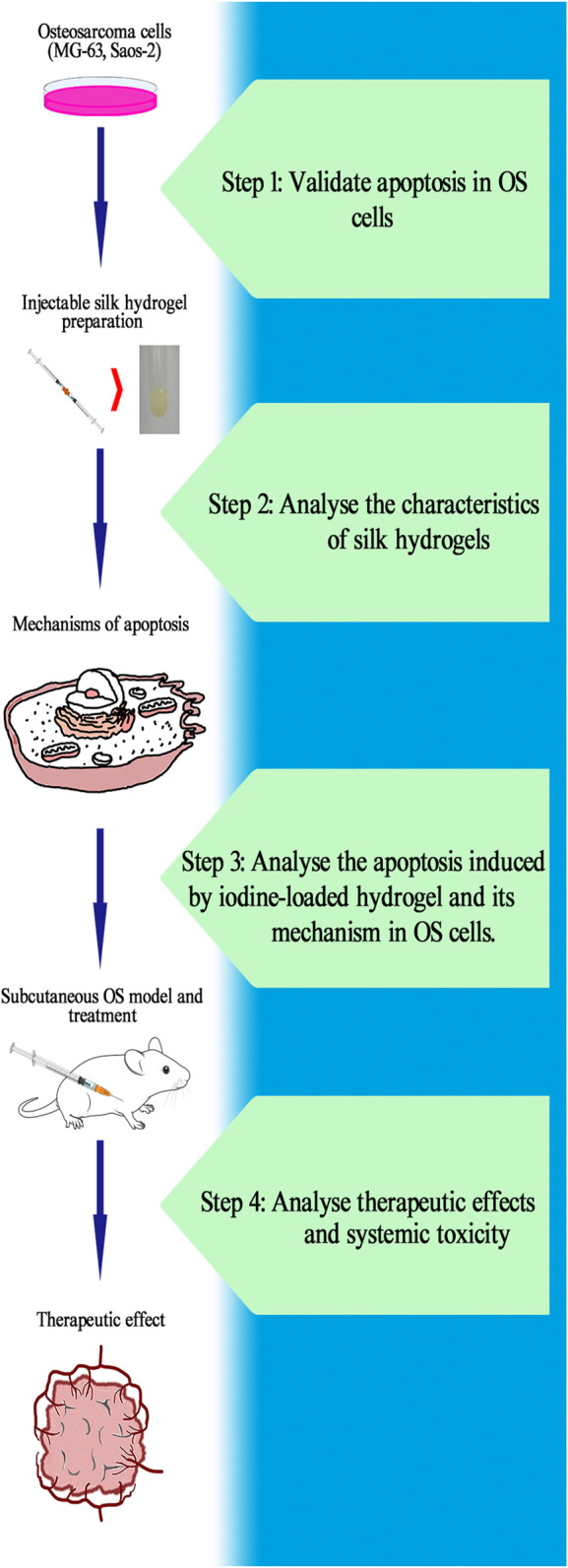
Ideal workflow for the research of self-assembling imageable silk hydrogels. Step 1: Verify the apoptosis induced by iodine in OS cells and calculate the IC_50_. Step 2: Prepare hydrogels and analyse its characteristics. Ensure that the hydrogels can be used as a controlled-release platform for iodine in the treatment of OS. Step 3: After treatment with released iodine solution, analyse the proportion of OS cells that undergo apoptosis, observe the apoptotic bodies and investigate the mechanism of apoptosis. Step 4: Construct a subcutaneous OS model and inject hydrogel into the OS to analyse the therapeutic effect and systemic toxicity of self-assembling imageable silk hydrogels on OS. (OS: osteosarcoma. IC_50_: the median inhibitory concentration).

Therefore, we developed a local drug delivery system for OS. An ideal workflow in this study is shown in Figure 1. We conducted *in vivo* experiments with subcutaneous OS models in nude mice. The subcutaneous OS model allows for the easy observation of OS growth and improves the feasibility of experimental surgery. The self-assembling imageable silk hydrogels can be radiologically imaged because they are mixed with MD. Hence, the hydrogel can be injected into the local OS site with X-ray guidance. Hydrogels can locally release iodine, which plays a role in OS therapy. Self-assembling imageable silk hydrogels allowed drug loading under general ambient conditions and led to sustained iodine delivery, suggesting that this approach is a promising avenue for focal OS therapy.

## Materials and Methods

### Cell Culture and Lentiviral Transduction

The human OS cell lines Saos-2 and MG-63 were purchased from the Shanghai Institute of Cell Biology, China. All cell lines were cultured in McCoy’s 5A (Sigma, United States) and MEM (Gibco, United States) supplemented with 10% foetal bovine serum (Gibco, United States) and 100 μg/ml normocin (InvivoGen, United States) at 37°C in 5% CO_2_. This study specifically used cells that were received less than 6 months prior to the beginning of the study. MG-63 cells were engineered with luciferase (LUC)-td tomato lentiviruses (GENE, China). Lentiviral production and concentration were performed according to standard procedures. MG-63 cells were transduced for 12 h at 37°C in 5% CO_2_. After 12 h, the cells were washed repeatedly to remove extracellular lentiviral particles, and the cells were cultured with 2 μg/ml puromycin to screen for luciferase-positive cells until all the cells in the control group had died. After screening with puromycin, MG-63 + LUC cells were harvested by using 0.25% trypsin containing 0.02% EDTA. A 100-μL cell suspension was seeded in a 96-well plate (8×10^3^ cells/well) and cultured for 24 h at 37°C in 5% CO_2_. After 24 h, the medium was removed, and 200 μL of fluorescein sodium solution (150 μg/ml) was added. The luciferase content in MG-63 cells (MG-63 + LUC) was measured with a Leica SP8 *in vivo* imaging system.

### Iodine Solution, Cell Counting Kit-8 (CCK-8) Assays, Flow Cytometry, and Transmission Electron Microscopy

An iodine solution containing 5 g/L I_2_ was prepared using polyvinyl pyrrolidone (PVP) (Sigma, United States), I_2_ (Sigma, United States) and deionized water. MG-63 and Saos-2 cells were washed three times with PBS and harvested by using 0.25% trypsin supplemented with 0.02% ethylenediaminetetraacetic acid (EDTA). A total of 100 μL of the cell suspension was seeded in a 96-well plate (5×10^3^ cells/well) and cultured for 24 h at 37°C in 5% CO_2_. After 24 h, the cells were divided into 11 groups (8 replicate wells/group). Different concentrations of iodine (0, 6, 8, 12, 14, 18, 20, 24 and 26 μM) and iodine solution released by self-assembling imageable silk hydrogels were added, and cell viability was measured at 3, 6, 12 and 24 h with Cell Counting Kit-8 solution (Dojindo, Japan). The optical density (OD) was measured at a wavelength of 540 nm using an Infinite 200Pro UV–visible spectrophotometer (Tecan, Austria). The differences in cell survival rates between the test group and the control group were compared. The median inhibitory concentration (IC_50_) was calculated. The cells were treated with iodine at the IC_50_ level in subsequent experiments. The following formula was used for calculations:

$$Cell\;survival\;rate\left(\%\right)=\frac{OD_{\left(treatment\;group\right)}-OD_{\left(blank\;group\right)}}{OD_{\left(control\;group\right)}-OD_{\left(blank\;group\right)}}\times 100\%$$

Apoptosis was assessed using the Annexin V-FITC/PI Apoptosis Detection Kit (BD, United States) according to the manufacturer’s specifications. After 12 h of treatment, MG-63 and Saos-2 cells were immediately collected and analysed using a fluorescence-activated cell sorting (FACS) flow cytometer (FACSCalibur, BD Biosciences, Heidelberg, Germany) for annexin V and PI staining. Annexin V+/PI + cells were considered the apoptotic cells.

For ultrastructural analyses to observe apoptotic corpuscles, after being washed with PBS, cells were fixed with 2% glutaraldehyde for 30 min and then further fixed with 1% (vol. ratio) osmium tetroxide for 2 h. Subsequently, the samples were dehydrated in an ethanol gradient and embedded in Spurr’s resin. The resin blocks were then cut into ultrathin sections (8 nm), and the sections were stained with uranyl acetate and lead citrate and examined using an electron microscope (Hitachi, Tokyo, Japan) operated at an accelerating voltage of 80 kV.

### Western Bloting

After the iodine solution released by the self-assembling imageable silk hydrogel and iodine treatment, human OS cells (Saos-2 and MG-63) were collected and the total protein was extracted and quantified using radioimmunoprecipitation assay (RIPA) buffer and a BCA protein assay kit (Beyotime, China). Total protein was boiled in buffer for 10 min, and equal amounts (30 μg) were resolved *via* 10 and 15% sodium dodecyl sulfate–polyacrylamide gel electrophoresis (SDS–PAGE) and transferred to polyvinylidene fluoride (PVDF) membranes (Bio–Rad). The membranes were blocked with skim milk dissolved in Tris-buffered saline (TBS) supplemented with 0.1% Tween-20, followed by incubation for 12 h at 4°C with the following primary rabbit polyclonal anti-human antibodies: GAPDH (1:1000, CST/5174, United States), Fas (1:1000, Proteintech/13098-1-AP, United States), FADD (1:1000, CST/2782, United States), FasL (1:1000, CST/68405, United States), caspase-3 (1:1000, Proteintech/19677-1-AP, United States), cleaved caspase-3 (1:1000, CST/9664, United States), PARP (1:1000, CST/9532, United States) and mouse monoclonal caspase-8 (1:1000, CST/9746, United States) antibodies. After three washes with TBS containing 0.1% Tween-20, the membranes were incubated with the appropriate secondary antibody for 1 h, washed three times, and visualized using enhanced chemiluminescence (ECL kit, Beyotime, China).

### Preparation of Injectable Silk Hydrogels

PEG 400 (MW 400 g mol^−1^), 60% silk fibroin solution, PVP-I (I_2_:5 g/L) and MD (Sigma, United States) were prepared. To make PEG-iodine-silk hydrogels, PEG was mixed with silk and PVP-I in 2.5 ml or 1 ml injectors. The total volume was 1 ml, and the percentage of each component in the PEG-silk hydrogel was as follows: 40% PEG 400 (V/V) and 13.5% (W/V) silk fibroin. The volume was adjusted to 1 ml with ultrapure water. The components of the PEG-silk/MD hydrogel were as follows: 40% PEG 400 (V/V), 13.5% silk fibroin (W/V), and 18% MD (V/V). The volume was adjusted to 1 ml with ultrapure water. The components of the PEG-iodine-silk/MD hydrogel were as follows: 40% PEG 400 (V/V), 13.5% silk fibroin (W/V), 18% MD (V/V), and 19.5% PVP-I (V/V). The volume was adjusted to 1 ml with ultrapure water.

### Characterization of the Silk Hydrogels

#### Gelation Time

After several solutions were mixed, the OD value also changed due to the change in structure. The gelation kinetics were recorded as a function of OD changes over time as reported previously (Matsumoto et al., 2006; Wang et al., 2015; Ma et al., 2016). The mixture was injected into a 96-well plate at 200 μL per well, and then, the plate was subjected to OD measurements at 550 nm in kinetics mode on an Infinite 200Pro UV–visible spectrophotometer (Tecan, Austria) set at 37°C. The OD change was monitored for more than 60 min at 1-min intervals. Each testing group contained 6 samples (*n* = 6) to obtain average OD values and standard deviations.

#### Scanning Electron Microscopy

PEG can complicate the freeze-drying process; thus, the hydrogels were rinsed to remove PEG 400 and then lyophilized. PEG can be removed when hydrogels are immersed in water. After the hydrogels were rinsed three times, they were frozen at −80°C overnight and lyophilized in a vacuum freeze drier (LICHEN LC-10N-50A, China) for 48 h. These freeze-dried gels were mounted on sample stubs and coated with Au, and images were captured using a scanning electron microscope (JEOL JSM-6300 SEM, Japan) at 12.5 kV (*n* = 3).

#### X-Ray Diffraction

A piece of hydrogel was evaluated by XRD (D/MAX-2500, Rigaku Co., Tokyo, Japan) to evaluate the degree of crystallinity. The samples were powdered and analysed with a 40-kV tube voltage, a 40-mA tube current, a 2θ = 5°–45° diffraction angle and a 4°/min scanning rate (*n* = 3).

#### Fourier Transform Infrared Spectroscopy

To determine whether the residual solvent and the addition of MD and PVP-I influence the conformational transformation, after removing PEG 400 and performing lyophilization, samples (*n* = 3) were powdered and placed on a zinc selenide (ZnSe) crystal cell, and the spectra were recorded in the reflection mode of the FTIR instrument (Thermo Fisher Scientific, Nicolet 6700 FT-IR,United States) in the spectral region of 550–4000 cm^−1^; background measurements were performed with an empty cell and subtracted from the sample reading. Deconvolution of the amide I spectra was performed using the Gaussian-Lorentzian function in Opus 4.2 software. (Bruker, United States).

#### Injectability

The injection performance of the hydrogel was evaluated by measuring the force required to inject a 200-μL sample from a 1-ml syringe through a 25-G 7/800 long needle with a crosshead speed of 114 mm min^−1^ corresponding to a 2 ml min^−1^ injection rate (Wang et al., 2015). PEG 400, silk solution, PVP-I solution and MD were mixed, and samples from each group were loaded into separate syringes and tested over 70 min. At the designated time, a syringe was selected and loaded into a custom fixture mounted on an Instron 3366 load frame (Instron, United States), and the entire sample was extruded. The data are presented as the average injection force (*n* = 3).

#### Mechanical Properties

Hydrogels (silk fibroin protein concentration of 13.5%) were prepared for mechanical tests, with an 8 mm final diameter and 10 mm height (*n* = 3). Samples were loaded in an Instron 5848 Microtester (Instron, United States) between stainless steel parallel plates, and the upper plate was lowered continuously at a rate of 1 mm/min until the compressive force did not increase due to gel fracture. The compressive stress and strain were determined by normalization against the sample geometries, and the elastic modulus was calculated as reported previously (Ma et al., 2016).

#### Iodine Release *in vitro*

The hydrogel samples (1 cm^3^) (*n* = 3) from each group were immersed in 4 ml of ultrapure water. The ultrapure water exposed to the hydrogel was collected, and the concentration of iodine released by each hydrogel was determined by the ICP–MS method. For the ICP–MS measurement of I, a matrix adjustment was necessary. For the measurements, the samples were diluted, and tetramethylammonium hydroxide was added, which caused hydrolysis of the organic compounds and decreased the redox potential. The liquid was then transferred into a corresponding centrifuge tube, and the samples were measured at 0, 3, 6, 9, 12, 24, 48, 72, 96, 120, 144 and 168 h. The release curve was generated according to the iodine release concentration measured by ICP–MS (Agilent 7700, United States).

### Osteosarcoma Xenografts in Nude Mice and Hydrogel Treatment

All animal experiments complied with animal research guidelines; the manuscript was written in accordance with the ARRIVE reporting guidelines, and the experiments were carried out according to the National Institutes of Health Guide for the Care and Use of Laboratory Animals (NIH Publications No. 8023, revised 1978). This study was performed in accordance with China’s national legislation and approved by the Ethics Committee of the First Affiliated Hospital of Harbin Medical University (ID:2021062), and the methods were conducted in accordance with the approved guidelines. Twenty 4-week-old female BALB/c athymic nude mice (14–15 g) were obtained from Beijing Vital River Laboratory Animal Technology Co., Ltd. According to the guidelines of the laboratory animal centre, the experimental animals were bred in a specific pathogen-free (SPF) environment in the experimental animal centre of Harbin Medical University with a constant temperature and humidity (22–24°C, 50–70% humidity) in a living environment under 12:12 h light:dark conditions. All animals were housed in pathogen-free conditions for an additional 7 days before OS inoculation. For the *in vivo* study, 100 μL of a suspension of 3 × 10^6^ MG-63 + LUC cells was subcutaneously injected into the right axilla of the mice. The OS volume was evaluated every 3 days using a vernier caliper, and the OS volume was calculated with the following formula: 1/6πab^2^ (a: long axis of the OS; b: short axis of the OS). When the OS volume was close to 0.5 cm^3^, the mice were randomly divided into four groups (5 mice per group). The control group was injected with normal saline, the PEG-silk/MD hydrogel group was injected with PEG-silk/MD hydrogels, the iodine group was injected with PVP-I and the PEG-iodine-silk/MD hydrogel group was injected with PEG-iodine-silk/MD hydrogels. The experimental group received an intratumoural injection of 50 μL of hydrogel and PVP-I, and the control group received an injection of 50 μL of saline. All operations were performed with X-ray guidance, and the mice were anaesthetized *via* intraperitoneal injection of 1% pentobarbital sodium (45 mg/kg) during the entire process. Then, 42 days after the injection, the animals were sacrificed by spinal dislocation under anaesthetization with intraperitoneal injection of 1% pentobarbital sodium (45 mg/kg), and the OSs and organs were harvested and fixed in 4% formalin.

### Live Bioluminescence Imaging

Forty-two days after the injection, the mice were anaesthetized *via* intraperitoneal injection of 1% pentobarbital sodium (45 mg/kg), and 200 μL of sodium fluorescein (Sigma, United States) (15 mg/ml) was injected intraperitoneally. After 10 min, *in vivo* imaging was performed with a Leica SP8 *in vivo* imaging system (Leica, GER).

### Histopathological Analysis

For the histopathological analysis, the collected OS samples and organs were fixed in 4% paraformaldehyde for 48 h at room temperature (RT) and then dehydrated using an alcohol gradient. The tissues were embedded in paraffin and cut into 4-μm sections. Sections were stained with haematoxylin and eosin (H&E) and analysed by immunohistochemistry (IHC). The paraffin-embedded tissue sections were mounted onto microscope slides, dewaxed and rehydrated. After hematoxylin and eosin (H&E) staining, images were taken under an optical microscope. For IHC staining, antigen retrieval was performed by microwave heating for 2 min in 0.1 mol/L citrate buffer (pH 6.0) followed by blocking with endogenous peroxide with 3% H_2_O_2_ in methanol for 5 min. After cooling, the slides were washed with distilled water and blocked with 10% goat serum for 30 min. The slides were then washed with distilled water and incubated with the primary antibodies anti-caspase-3 (1:200, Proteintech/19677-1-AP, United States) and anti-PARP(1:200, CST/9532, United States) at 4°C for 12 h and then rinsed with running water and PBST. The slides were then stained with the secondary antibody goat anti-rabbit IgG H&L (Abcam, United States) for 60 min, washed in running tap water, stained with diaminobenzidine (DAB), and counterstained with haematoxylin. Finally, the slides were dehydrated and visualized using a microscope. The results were analysed with Image-Pro Plus (IPP) 6.0 software (Media Cybernetics, United States).

### Statistical Analysis

All statistical analyses were performed using SPSS 19.0 (SPSS, Inc., Chicago, IL, United States). When the data were normally distributed, two samples were analysed using Student’s t test to determine significant differences (*p* ≤ 0.05), and multiple comparisons were evaluated by one-way analysis of variance (ANOVA) followed by Dunnet’s test to determine whether there were significant differences (*p ≤ 0.05, **p ≤ 0.01, ***p ≤ 0.001). Continuous variables are expressed as the mean ± standard deviation. During data analysis, the analysts were unaware of the groups and treatments.

## Results

### Iodine Induces the Apoptosis of OS Cells

**FIGURE 2:**
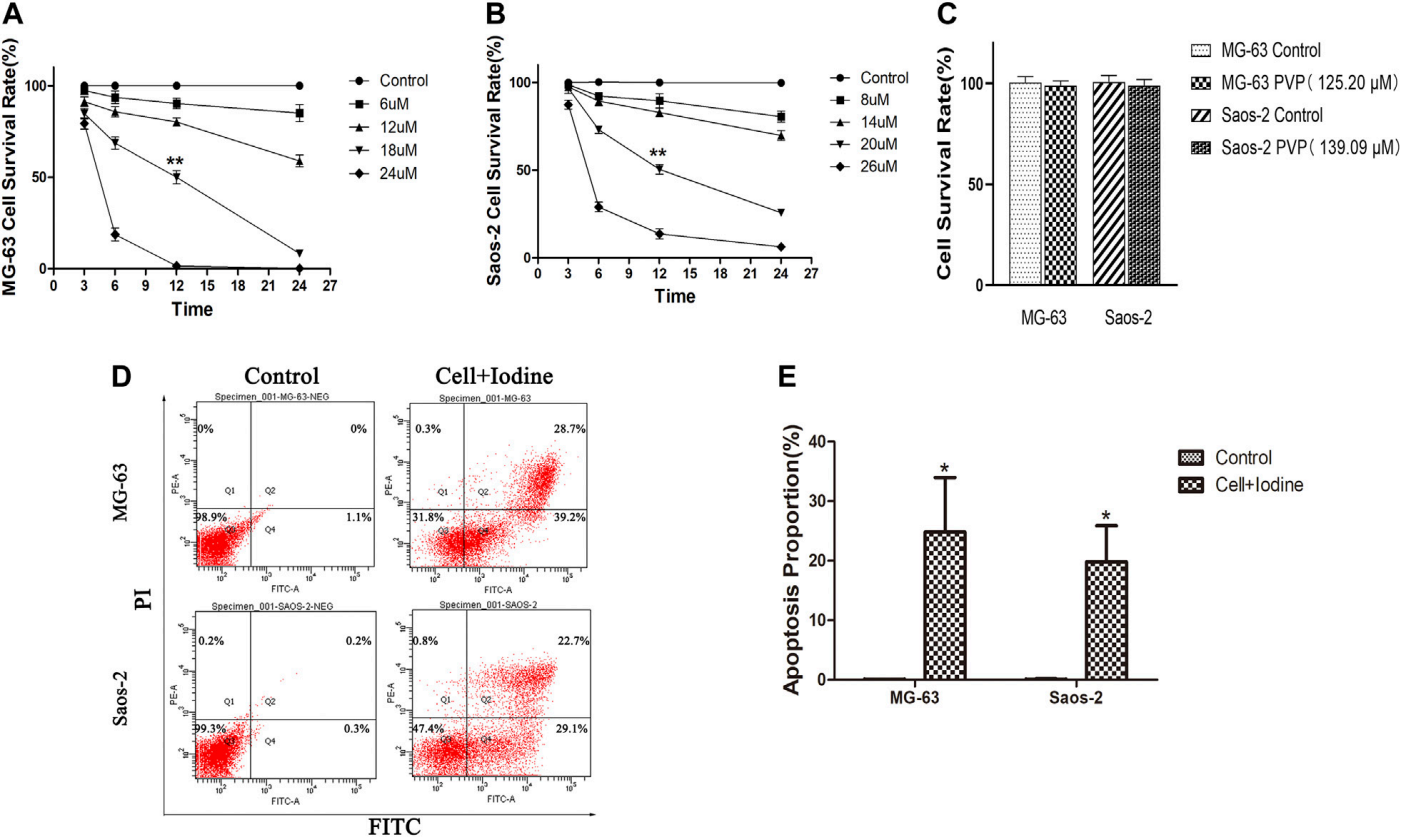
Iodine induced apoptosis in OS cells. **(A,B)** After 3, 6, 12 and 24 h of treatment with iodine, the cell survival rate was assessed using a CCK-8 assay. The IC_50_ values of MG-63 and Saos-2 at 12 h were 18 and 20 μM, respectively (**p < 0.01). **(C)** The cell survival rate of MG-63 and Saos-2 cells after treatment with PVP solution (125.20 and 139.09 µM) for 12 h, which corresponded to the iodine (18 and 20 µM) concentration used. **(D)** After 12 h of treatment with iodine, the proportions of cells (MG-63 and Saos-2) undergoing apoptosis were analysed by flow cytometry after staining with Annexin V-FITC/PI. **(E)** The proportion of apoptotic OS cells after treatment with iodine (*p < 0.05). The mean percentages of cells undergoing apoptosis (±SD) are shown. (OS, osteosarcoma; CCK-8, Cell Counting Kit-8; IC_50_, the median inhibitory concentration; PVP, polyvinylpyrrolidone; Annexin V-FITC/PI, Annexin V-FITC/PI Apoptosis Detection Kit; SD, standard deviation).

We analysed the effect of iodine on the survival rates and apoptosis of MG-63 and Saos-2 OS cells. As shown in Figures 2A,B, treatment of MG-63 and Saos-2 cells with PVP-I stimulated cell death with time- and concentration-dependent effects, respectively. When the iodine concentrations were 18 and 20 μM, the cell survival rates were 50 ± 2.1% and 50.5 ± 2.7% for MG-63 and Saos-2 cells at 12 h, respectively (*p* < 0.01). We identified the I_2_ concentration at 12 h as the IC_50_ value. The PVP concentrations corresponding to iodine concentrations of 18 and 20 µM were 125.20 and 139.09 µM, respectively. PVP solution was also used to stimulate the two types of cells for 12 h and did not affect cell survival (Figure 2C), which suggested that iodine causes cell death.

As the flow cytometry results showed, after 12 h of treatment with 18 and 20 µM iodine, the proportions of MG-63 and Saos-2 undergoing apoptosis were 24.8 ± 9.2% and 19.8 ± 6.1%, respectively, which were significantly higher than those of the control cells (*p* < 0.05) (Figures 2D,E).

### Characterization of the Silk Hydrogels

#### Gelation Time

**FIGURE 3:**
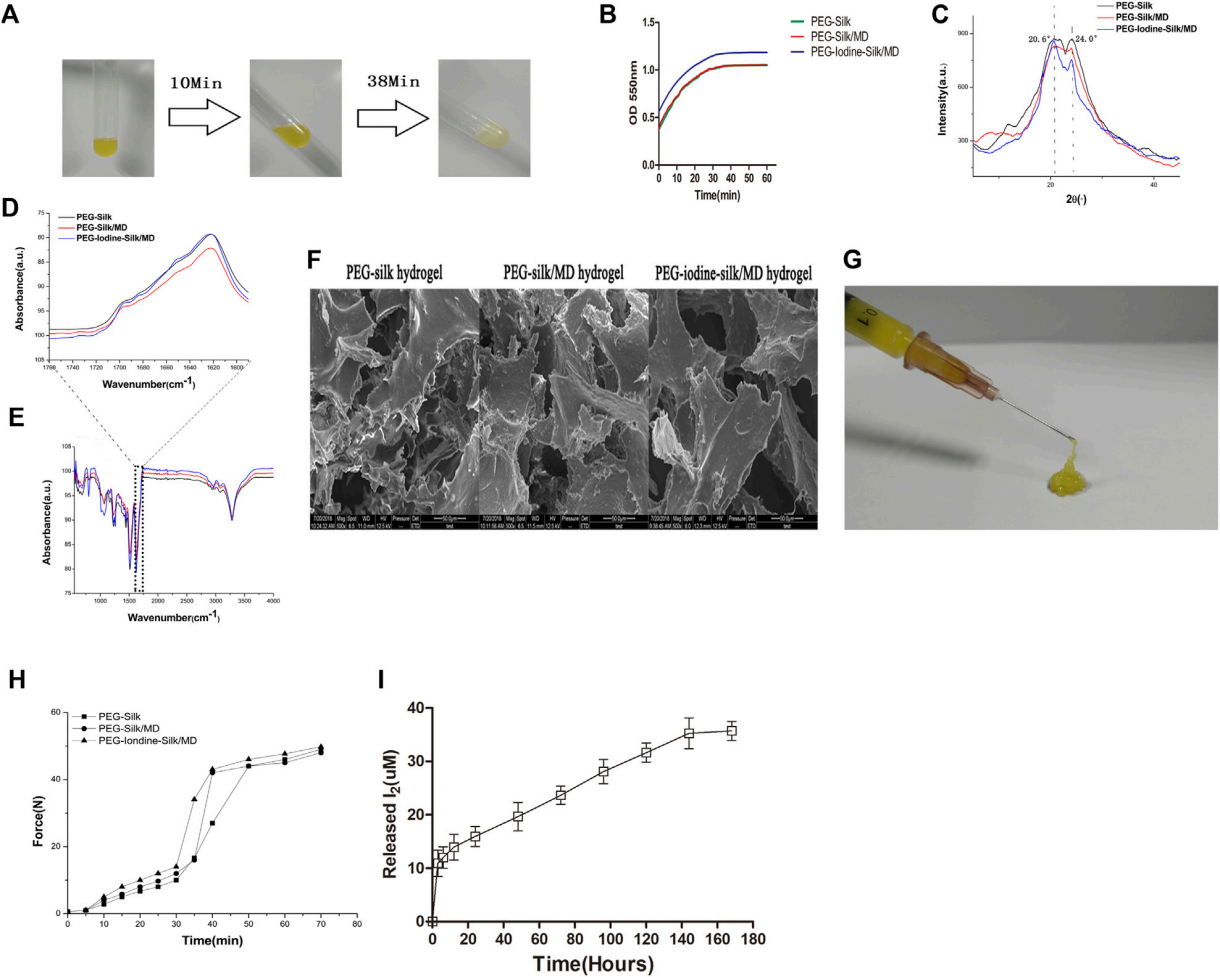
Characterization of the silk hydrogels. **(A)** Gelation process after mixing silk fibroin, PEG 400, MD and PVP-I solution at room temperature. **(B)** Time-dependent OD measurement after mixing silk fibroin, PEG 400, MD and PVP-I solution. **(C)** XRD measurement of the silk hydrogels. **(D,E)** FTIR spectra of the PEG-silk hydrogel, PEG-silk/MD hydrogel and PEG-iodine-silk/MD hydrogel. **(F)** Silk hydrogel morphologies determined by SEM (scale bars = 50 µm). **(G,H)** Injectability measurement. **(I)** I_2_ released from the PEG-iodine-silk/MD hydrogel. (PEG 400, polyethylene glycol 400; MD, meglumine diatrizoate; PVP-I, polyvinylpyrrolidone iodine; OD, optical density; XRD, X-ray diffraction; FTIR, Fourier transform infrared spectroscopy; PEG, polyethylene glycol; SEM, scanning electron microscopy).

The solutions were fluids when the ingredients were mixed and became more viscous and opaquer over time (Figure 3A). The gelation time was defined as the time from the beginning of mixing to the time when the plateau was reached. As shown in Figure 3B and Table 1, the gelation times of the three groups ranged from 36.7 min to 37.7 min. The gelation time depended on the concentrations of silk and PEG used. The similarity of the gelation time between the three groups was due to the use of the same concentrations of silk and PEG 400 in all of the gels.

**TABLE 1:** Silk gelation time.

Concentrations of silk (W/V, %) and PEG 400 (V/V, %)	PEG-silk hydrogel (min)	PEG-silk/MD hydrogel (min)	PEG-Iodine-silk/MD hydrogel (min)
13.5/40	36.7 ± 0.58	37.3 ± 0.58	37.7 ± 1.15

The gelation time was defined as the time when the optical density plateaued (*n* = 6). There was no significant difference in gelation time among the three groups (*p* > 0.05) (PEG: polyethylene glycol, MD: meglumine diatrizoate).

#### Crystallinity Analysis by XRD

XRD was performed to study the crystallinity of the silk hydrogels. The XRD results revealed analogous crystallization in all three types of hydrogels. A strong peak at 20.6° implied that the silk existed mainly in the silk II form (Figure 3C), further confirming the FTIR results. The results indicated that PVP-I and MD had a negligible influence on the silk crystallinity analysis.

#### Structural Analysis by FTIR

Silk gelation was accompanied by *β*-sheet structure formation, as shown by the major peak at 1622 cm^−1^ in the amide I region of the FTIR spectrum (Figure 3D). The strong peak at wavelengths lower than 1218–1262 cm^−1^ and the peak centred at 2934–2952 cm^−1^ resulted from residual PEG (Figure 3E), as reported in the literature (Nguyen and Janik, 1987). PEG did not show peaks in the amide I and II band regions and thus had a negligible influence on the silk structural analysis. As shown in Figures 3D,E, PVP-I and MD did not affect the silk structure.

#### Microstructure of Lyophilized Silk Hydrogels

The porous morphology of 13.5% silk hydrogels after extraction and lyophilization was examined by SEM. The overall gel morphology showed small pores, an elongated shape and randomly orientated silk laminar layers, indicating high porosity and interconnectivity. The diameter of the pores, whose walls were extraordinarily thin, varied from 20 to 100 µm (Figure 3F, Supplementary Figure S1). The presence of PEG might have changed the freezing rate and the pore structure of the silk gels during lyophilization. Since the concentrations of silk and PEG were the same in all three groups, all of the hydrogels had similar pore diameters.

#### Mechanical Properties and Injectability of Silk Fibroin Hydrogels

The hydrogels were subjected to an unconfined compressive strain to the point of failure to assess their mechanical properties. The compressive strengths, elastic moduli, and strains-to-failure of the hydrogels were similar (Table 2) because the concentrations of silk fibroin and PEG were the same in all three groups. The gels were easily injected with a syringe (Figure 3G). The supervised injection force started to significantly increase 30–40 min after mixing the solutions. The injection forces reached 50 N by the end of the measurement period (60–70 min) and continued to slowly increase afterwards (Figure 3H). These properties contributed to the ease of hydrogel injection.

**TABLE 2:** Unconfined compression data for silk hydrogel.

Group	PEG-silk hydrogel	PEG-silk/MD hydrogel	PEG-Iodine-silk/MD hydrogel
Compressive strength (kPa)	15.7 ± 0.8	16.1 ± 0.9	16.5 ± 0.8
Elastic modulus (kPa)	146.5 ± 1.5	150.0 ± 2.0	151.67 ± 2.46
Strain-to-failure (%)	14.17 ± 0.35	14.9 ± 0.4	14.97 ± 0.35

The concentrations of silk (w/v, %) and PEG, 400 (w/w, %) were 13.5 and 40%, respectively (*n* = 3). The results showed that there was no significant difference in unconfined compression among the three groups (*p* > 0.05) (PEG, polyethylene glycol; MD, meglumine diatrizoate).

#### Iodine Release

Figure 3I shows the quantity of iodine released into the medium at different time points. The quantity of iodine released increased over time. The results showed a burst release in the first 12 h; then, the release of iodine slowed and sustained release occurred over the following 6 days. The concentration of released iodine reached its highest level of 35.3 μM on day 7. Furthermore, when the concentration of released iodine was highest, the cumulative release amount following 7 days of release was approximately 36.3% of the initial iodine content.

### Iodine Solution Released by Self-Assembling Imageable Silk Hydrogels Induces the Apoptosis of OS Cells

**FIGURE 4:**
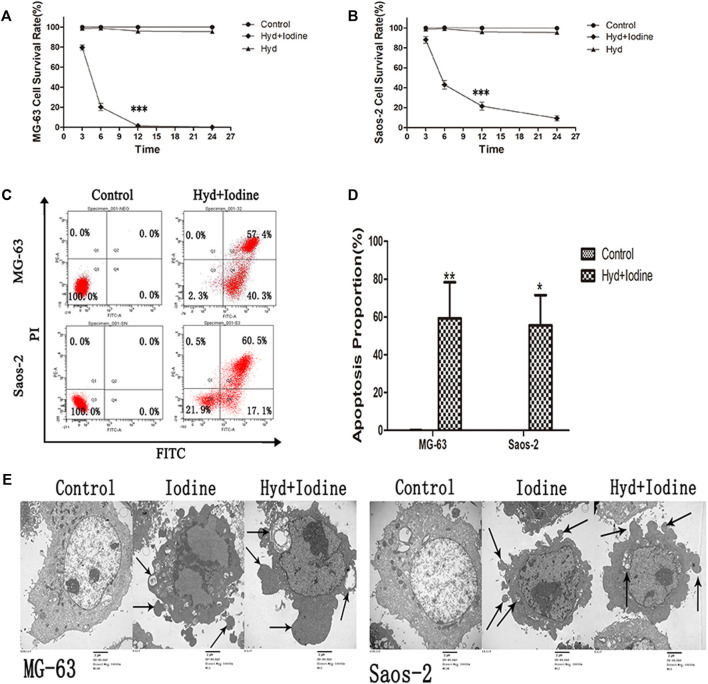
Iodine released by the self-assembling imageable silk hydrogel induced apoptosis in OS cells. **(A,B)** After 3, 6, 12 and 24 h of treatment with released iodine solution, the cell survival rate was assessed by a CCK-8 assay (***p < 0.001). **(C)** After 12 h of treatment with released iodine solution, the proportions of cells (MG-63 and Saos-2) undergoing apoptosis were analysed by flow cytometry after staining with Annexin V-FITC/PI. **(D)** The proportion of apoptotic OS cells after treatment with released iodine solution (*p < 0.05, **p < 0.01). The mean percentages of cells undergoing apoptosis (±SD) are shown. **(E)** The apoptotic ultrastructure of OS cells treated with iodine and released iodine solution. The arrows indicate apoptotic bodies. The cells were examined under a transmission electron microscope. Control: untreated OS cells, Iodine: treated with PVP-I, Hyd + Iodine: treated with iodine solution released by hydrogel (Magnification, 10000X; Scale bar = 2 µm) (OS, osteosarcoma; CCK-8, Cell Counting Kit-8; Annexin V-FITC/PI, Annexin V-FITC/PI Apoptosis Detection Kit; SD, standard deviation; PVP-I, polyvinylpyrrolidone iodine).

The anti-OS ability of the self-assembling imageable silk hydrogels was verified *in vitro*. The hydrogel (1 cm^3^) was immersed in 4 ml of OS cell culture medium, and the cells were cultured in medium for 12 h. We analysed the effect of released iodine solution on the survival rates of OS cells. To verify the effects of both types of OS cells on apoptosis at the same time, with reference to the iodine release results (Figure 3I), we chose the iodine solution released by the hydrogel for 72 h. As shown in Figures 4A,B, the anti-OS effect of the self-assembling imageable silk hydrogels was excellent. The cell survival rates were 2.0 ± 0.4% and 20.3 ± 3.5% for MG-63 and Saos-2 cells at 12 h, respectively, which were significantly lower than those of the control group and the hydrogel group without iodine loading (*p* < 0.001).

To further verify the occurrence of apoptosis, we observed the morphology of dead cells by flow cytometry and TEM. After 12 h of released iodine solution and iodine treatment, the proportions of cells undergoing apoptosis were 59.3 ± 19.1% and 55.6 ± 15.9% (Figures 4C,D), respectively. The apoptosis rates of the MG-63 and Saos-2 cells were significantly higher than those of the control cells (*p* < 0.05) (Figure 4D). Typical morphological features of apoptosis were observed by TEM, including the following: condensation of nuclear chromatin, denser cytoplasm, and the formation of dense round apoptotic bodies (Figure 4E). Plasma membrane blebbing and shedding of apoptotic bodies indicated apoptotic cell death. These data demonstrated that iodine solution released by the hydrogel and iodine alone enhanced MG-63 and Saos-2 cell apoptosis *in vitro*.

### Iodine Induces Saos-2 and MG-63 Cell Apoptosis by Regulating the Fas/FasL Signalling Pathway

**FIGURE 5:**
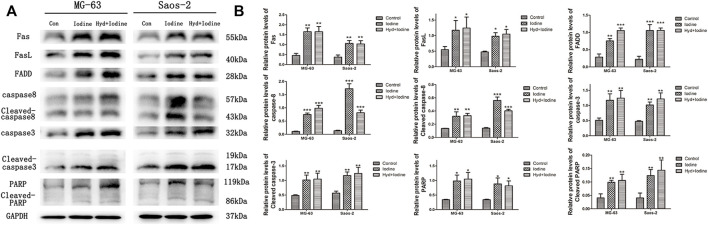
Iodine induces apoptosis through the death receptor pathway. **(A)** A representative Western blot of Fas, FasL, FADD, caspase-8, cleaved caspase-8, caspase-3, cleaved caspase-3, PARP and cleaved PARP in protein extracts of MG-63 and Saos-2 cells pretreated with iodine and released iodine solution for 12 h. **(B)** Densitometric analysis of Fas, FasL, FADD, caspase-8, cleaved caspase-8, caspase-3, cleaved caspase-3, PARP and cleaved PARP levels. The results are presented as the mean ± SD (*n* = 3) (*p < 0 0.05, **p < 0 0.01, ***p < 0 0.001) (SD: standard deviation).

To further elucidate how iodine causes apoptosis, we performed Western blot analysis. The Western blot results showed that the levels of Fas, FasL and FADD were significantly increased in MG-63 and Saos-2 cells after incubation with iodine and released iodine solution compared with those of the control group. Similarly, the levels of caspase-8, caspase-3, PARP and their cleaved forms were also increased in both MG-63 and Saos-2 cells after treatment with iodine and released iodine solution compared with those of the control group (Figure 5A). In the quantitative analysis, the expression levels of Fas (40–55 kDa), FasL (40 kDa), FADD (28 kDa), caspase-8 (57 kDa), cleaved caspase-8 (43 kDa), caspase-3 (32 kDa), cleaved caspase-3 (17/19 kDa), PARP (116 kDa) and cleaved PARP (89 kDa) were significantly higher in iodine-treated cells than in control cells (Figure 5B) (*p* < 0.05).

### Iodine Inhibition of OS Growth *In Vivo* in a Nude Mouse Model

**FIGURE 6:**
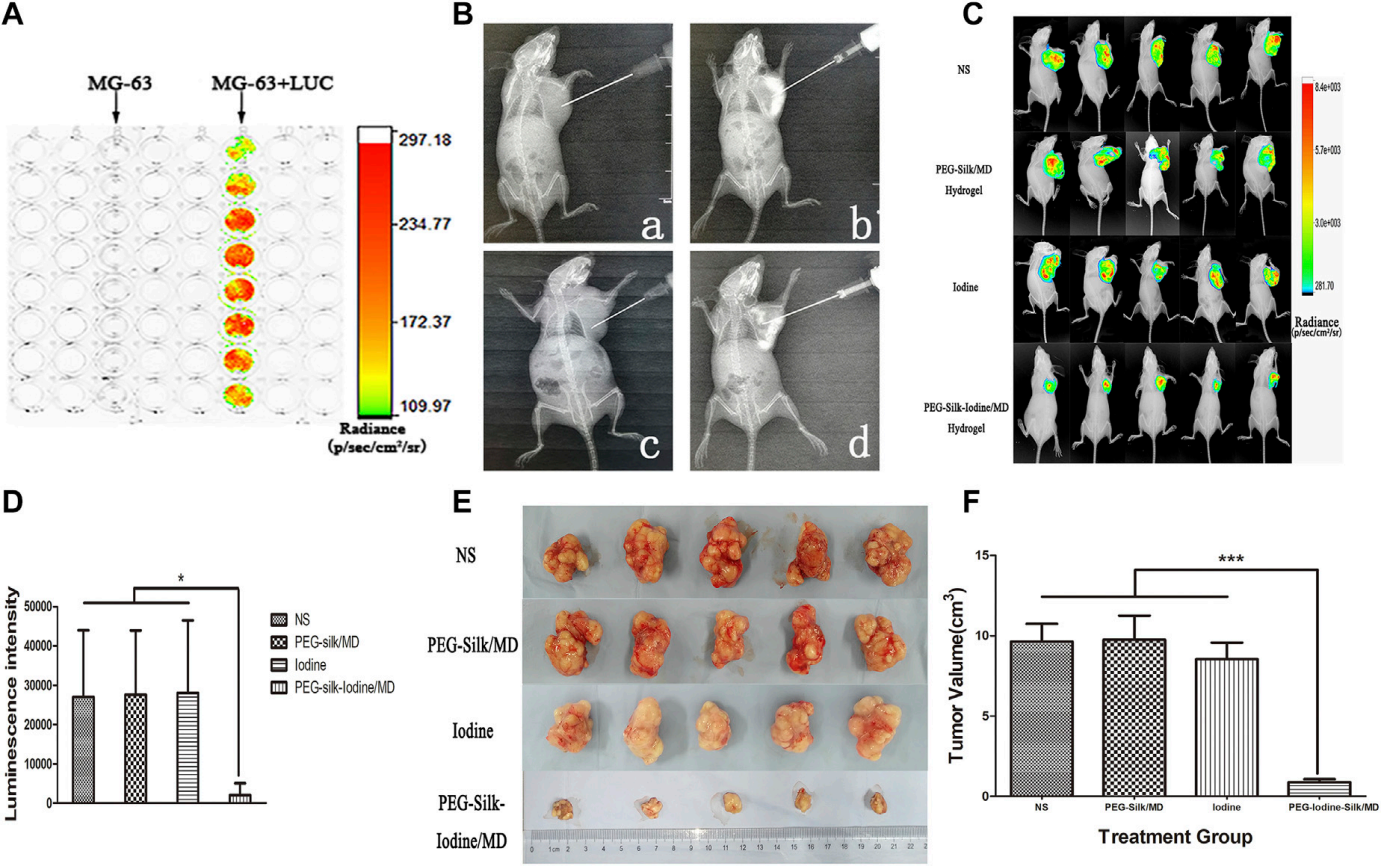
Iodine-loaded hydrogels inhibit OS growth *in vivo*. **(A)** The bioluminescence of MG-63 cells after transfection with luciferase. **(B)** A representative image of the injectable hydrogels in the X-ray field. The image shows the distribution of the imageable hydrogels in the OS. a, Control group mice were inoculated with normal saline. b, PEG-silk/MD hydrogel group mice were inoculated with PEG-silk/MD hydrogel. c, Iodine group mice were inoculated with PVP-I. d, PEG-iodine-silk/MD hydrogel group mice were inoculated with PEG-iodine-silk/MD hydrogel. **(C)** The bioluminescence imaging of OS *in vivo*. **(D)** Quantification of bioluminescence signals from OS-bearing mice. **(E)** A representative image of xenograft OSs in the four groups. **(F)** Volume of xenograft OSs in nude mice after 42 days of hydrogel and saline injection. The data are presented as the mean ± SD. (*p < 0.05, ***p < 0.001.) (OS, osteosarcoma; PEG, polyethylene glycol; MD, meglumine diatrizoate; PVP-I, polyvinylpyrrolidone iodine;SD, standard deviation).

The effect of iodine on OS was explored *in vivo*. Fluorescence was observed after the successful transfection of MG-63 cells with luciferase (LUC)-td tomato lentiviruses (Figure 6A). Silk hydrogels contain MD; therefore, the injection, distribution and dispersion of the silk hydrogel can be visualized by X-ray analysis (Figure 6B). For *in vivo* bioluminescence imaging, the bioluminescence levels in the PEG-silk-iodine/MD hydrogel-treated mice decreased significantly compared with those in the control, iodine and PEG-silk/MD hydrogel mice (*p* < 0.05) (Figures 6C,D), whereas the bioluminescence levels in the mice that were treated with PVP-I and PEG-silk/MD hydrogel were not decreased compared with those in the control mice. As shown in Figures 6E,F, the OS volume in the group of mice injected with PEG-iodine-silk/MD hydrogel was significantly smaller than that in the other three groups of mice (*p* < 0.001). These results indicated that OS growth was significantly inhibited in the mice that received iodine released from the hydrogel. In the pre-experiments we constructed animal models using both MG-63 + LUC and Saos-2+LUC cells, but we found that Saos-2 cells could not form subcutaneous tumours (Supplementary Figure S2).

### Immunohistochemical Staining and Systemic Toxicity

**FIGURE 7:**
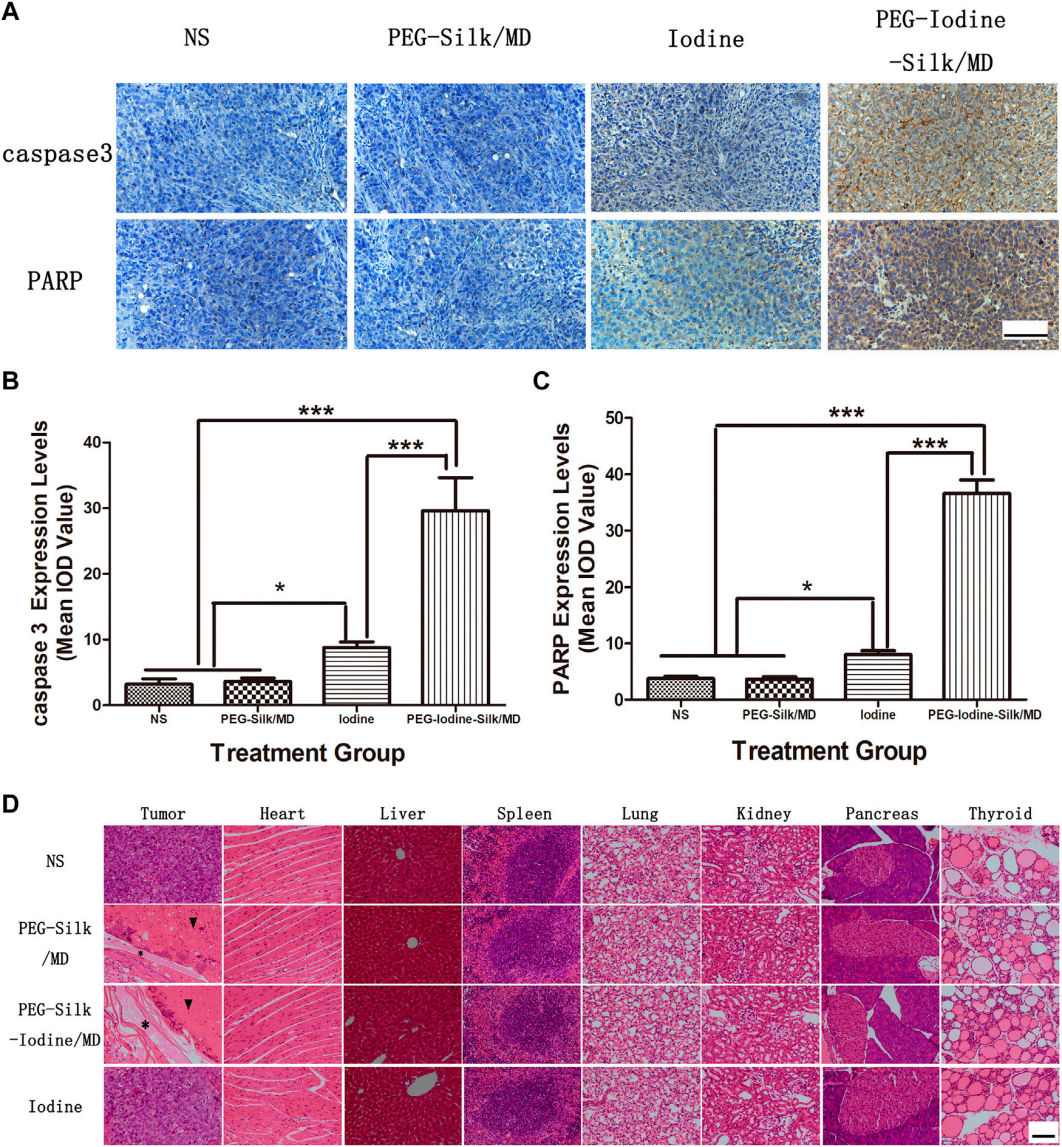
Immunohistochemical and H&E staining. **(A)** Caspase-3 and PARP expression in OS sections from the different groups was characterized by immunohistochemistry. The data are representative images (scale bars = 100 µm). **(B)** Quantification of the caspase-3 staining densities. The data expressed as the mean ± SD of in individual groups of OSs (*n* = 5) (*p < 0.05, ***p < 0.001). **(C)** Quantification of the PARP staining densities. The data are expressed as the mean ± SD of individual groups of OSs (*n* = 5) (*p < 0.05, ***p < 0.001). **(D)** OSs and main organs (heart, liver, spleen, lung, kidney, pancreas and thyroid) were dissected for H&E staining on the 42nd day after injection. No significant abnormal damage was observed in the organs. * indicates silk hydrogel. ▾ indicates necrotic OS tissue (scale bars = 100 µm). (OS, osteosarcoma; PEG, polyethylene glycol; MD, meglumine diatrizoate; SD, standard deviation; H&E, haematoxylin and eosin; IOD, integrated optical density).

The levels of caspase-3 and PARP expression are associated with the apoptosis in OS. We used IHC to measure the expression levels of caspase-3 and PARP in different OS groups (Figure 7A). Quantitative analysis of the intensity of anti-caspase-3 and anti-PARP staining indicated that there were no significant differences in OS between the control and PEG-silk/MD hydrogel groups (Figure 7A). The levels of caspase-3 and PARP expression in the PEG-iodine-silk/MD hydrogel group were significantly higher than those in the control, iodine and PEG-silk/MD hydrogel groups (*p* < 0.001), whereas the levels of caspase-3 and PARP expression in the mice that were treated with PVP-I were increased compared with those in the control and PEG-silk/MD hydrogel mice (*p* < 0.05) (Figures 7B,C). Collectively, these data indicated that treatment with the implanted PEG-iodine-silk/MD hydrogel increased the expression levels of caspase-3 and PARP in the OS model in nude mice. Furthermore, we examined the biocompatibility and systemic toxicity of the silk hydrogels. At day 42 after injection, the mice were sacrificed, and important organs in each group were collected, including the heart, liver, spleen, lungs, kidneys, pancreas and thyroid. No cells with tumour cell characteristics were observed in the organs; in particular, the thyroid, which is sensitive to iodine, was unaffected, which is critical, as changes in iodine levels often cause pathological changes in the thyroid (Figure 7D). This result indicated that the PEG-iodine-silk/MD hydrogel exhibited good biocompatibility and biosafety *in vivo*. In addition, as shown in Figure 7D, incompletely degraded hydrogel and a large amount of necrotic OS tissue appeared around the PEG-iodine-silk hydrogel (* indicates silk hydrogel; ▾ indicates necrotic OS tissue).

## Discussion

In this study, we showed that iodine significantly inhibited proliferation and induced apoptosis in OS cell lines. The inhibitory effect of iodine on cell proliferation was time- and concentration-dependent (*p* < 0.01). In the flow cytometric analysis, the rates of MG-63 and Saos-2 cells undergoing apoptosis were approximately 24.8 ± 9.2% and 19.8 ± 6.1% after 12 h of iodine stimulation at 18 and 20 μM, respectively, and the apoptosis rates were significantly higher in the OS cells than in the control cells (*p* < 0.05). We also used TEM to observe typical apoptotic morphological features, including the condensation of nuclear chromatin, denser cytoplasm, and the formation of dense round apoptotic bodies (Li et al., 2015; Antunovic et al., 2019). These results indicated that iodine induced apoptosis in MG-63 and Saos-2 cells.

Although iodine has been widely used in antitumour studies, its antitumour mechanism is still unclear. In this study, we elucidated the mechanism by which iodine activates the death receptor pathway, leading to apoptosis in OS cells. We found that apoptotic cell death in OS cells (MG-63 and Saos-2) was predominantly initiated by the death receptor Fas (40–55 kDa), which bound to FasL (40 kDa) to activate the death domain FADD (28 kDa); subsequently, caspase-8 (57 kDa) was activated (Nagata, 1997; Ashkenazi and Dixit, 1998; Kuang, et al., 2000). The first cleavage stabilizes the active dimer, and the second cleavage releases it from the death-induced signalling complex (DISC) (Dickens et al., 2012; Yin et al., 2018; Wu et al., 2019). In our study, cleaved caspase-8 (43 kDa) activated caspase-3 (32 kDa), which further activated PARP to promote apoptosis (Green and Llambi, 2015). PARP is a 116 kDa nuclear poly(ADP-ribose) polymerase, and cleaved PARP contains the carboxy-terminal catalytic domain (89 kDa) (Lazebnik et al., 1994; Nicholson et al., 1995). The cleavage of PARP facilitates DNA fragmentation and cell disintegration and disassembly, thus serving as a marker of cells undergoing apoptosis (Oliver et al., 1998).

Silk fibroin is known to be degraded *in vivo* and has a robust safety record in humans (Leisk et al., 2010; Omenetto and Kaplan, 2010; Diab et al., 2012; Ma et al., 2016; Leng et al., 2017). The degradation profile of silk hydrogels can be controlled and can last from weeks to months depending on the crystallinity level (Vepari and Kaplan, 2007). Additionally, different forms of silk fibroin products, such as microparticles, films and hydrogels, can allow stabilization of the drug load and control of drug release using endogenous parameters such as the crystallinity and molecular weight of the silk fibroin (Pritchard and Kaplan, 2011; Wenk et al., 2011; Li et al., 2016). Although numerous silk formulations have been used in previous studies, no study has examined the therapeutic potential of silk hydrogels for human OS therapy *in vitro* or *in vivo*. In this study, we developed a local delivery system that was injectable, imageable, biodegradable and safe and was capable of delivering anti-OS drugs that target OS cells *in vitro* and *in vivo*. In previous studies, high silk fibroin concentrations (>8%) were suggested to be desirable to avoid rapid *in vivo* degradation of the gel (Wang et al., 2015). In our study, silk fibroin concentrations reached 13.5%, which ensured slow *in vivo* degradation of the gel. Only low-molecular weight PEG (<1500 Da) can force the hydrophobic domains on the silk molecules to form crystalline *β*-sheets and a gel network; in contrast, higher-molecular weight PEG induces silk to form fibres and particles (Wang et al., 2015). Regarding the use of PEG, as a pharmaceutical ingredient, the highest concentration of PEG 400 used in medicinal formulations for injection approaches 50% (Strickley, 2004). In our study, the concentration of PEG 400 in the silk hydrogels was 40% (<50%); thus, toxicity was not a concern.

This study aimed to explore the feasibility of using imageable hydrogels to deliver anti-OS drugs for the treatment of OS. We analysed the hydrogel characteristics. During the gelation of silk fibroin, nanospheres, nanofilaments and microfibres formed, which affected the clarity of the solution. Therefore, the change in the OD could be measured to monitor the molecular hierarchical self-assembly process during silk gelation. In our study, the gelation time was 36.7–37.7 min. The change in the injection force was also determined. The retardation times measured by the change in the injection force (40 and 50 min) were longer than those measured by the change in the OD (37 min). This difference may be due to the difference in the testing temperature, as the injection experimental temperature (RT) was lower than that used in the gelation time experiment (37°C). Another possible reason is that the molecular entanglements of silk such that these structural changes preceded the formation of nano- and microstructural features, resulting in a time discrepancy between force and OD measurements. In addition, the injection of a mixed solution required less than a 10 N compression force to pass through a thin needle (27 G). At the end of the measurement, the force was 40–50 N, but the hydrogel was still injectable despite requiring more force. A relatively slow increase in injection force and smaller compression force could also be observed for silk hydrogels, which could be beneficial for clinical application. As shown by scanning electron microscopy, the internal network of the gel was small and compact. In addition, the mechanical properties of the three hydrogels were stable and homoplastic. The process of hydrogel formation is also a process of *β*-sheet and gel network formation. The main diffraction peaks found at 12.3°, 19.7°, 24.7°, 28.5° and 33.3° represented silk I, while those at 9.1°, 18.8° and 20.5° represented silk *β*-sheet structures.The curves that had absorption bands in the frequency range of 1620–1630 cm^−1^ and 1695–1700 cm^−1^ represented an enriched *β*-sheet structure in the silk II form (Wang et al., 2015). The contribution of these curves (β-sheet structural content) to the amide I band was assessed by integrating the area under the curve and then normalizing it to the total area under the amide I band region (1600–1700 cm^−1^), as described previously. Although mixed with PVP-I and MD, based on XRD and FTIR, PVP-I and MD had no obvious impact on the conformation transition because they did not interact with silk or PEG; only phase separation was observed. Silk hydrogels are a mature and stable drug release system; nevertheless, we tested silk hydrogels for their ability to release iodine. As the results showed, in the first 12 h, iodine was released quickly. The release of iodine subsequently slowed and was sustained over 6 days, maintaining a maximum concentration of 35.3 μM. Furthermore, the cumulative released amount following 7 days of release was approximately 36.3% of the initial iodine content. We chose the iodine solution released by the hydrogel for 72 h, and treated MG-63 and Saos-2 for 12 h, the proportions of cells undergoing apoptosis were 59.3 ± 19.1% and 55.6 ± 15.9%, respectively. The apoptosis rates of the MG-63 and Saos-2 cells were significantly higher than those of the control cells (*p* < 0.05). This indicate that the iodine released from self-assembled hydrogels can effectively promote MG-63 and Saos-2 apoptosis *in vitro*.

With X-rays, the imageable hydrogels were observed as a high-density image, and X-rays could be used to guide the localization of the hydrogels injected into the OS. As the X-ray image shows, the hydrogels remained inside the OS and dispersed. With the introduction of MD and imaging in the X-ray field, the injection was less invasive, which was beneficial for injecting hydrogels and delivering anti-OS drugs to local lesions.

We then determined whether our *in vitro* observations would translate into *in vivo* OS therapy. Therefore, we used a humanized subcutaneous OS nude mouse model. In the *in vivo* study, the local treatment of mice with PEG-iodine-silk/MD hydrogels suppressed OS growth. Compared with the other three groups, the group treated with PEG-iodine-silk/MD hydrogels showed not only a significantly decreased OS volume but also a significant decrease in luminescence intensity. Immunohistochemical tests were performed to determine whether iodine transferred by hydrogels *in vivo* could induce apoptosis as it did *in vitro*. We found that the expression levels of caspase-3 and PARP in the PEG-iodine-silk/MD hydrogel treatment group were significantly higher than those in the other three groups (*p* < 0.001). These results indicated that iodine can induce the apoptosis of OS *in vivo* and *in vitro*. The local injection of PVP-I into the OS did not achieve ideal results. We suspected that the injection of PVP-I alone, without hydrogel to control its release, could cause iodine to enter the circulation system quickly and be metabolized without long-term effects on OS. The systemic damage caused by iodine was far less severe than that caused by chemotherapeutic drugs. The results show that the iodine-loaded hydrogels had high biological safety and no systemic toxicity and induced no damage to the main organs, such as the heart, liver, spleen, lung, kidneys, pancreas and thyroid. In particular, thyroid tissue is sensitive to iodine, and changes in iodine can cause thyroid tissue lesions. However, in this study, iodine-loaded hydrogels did not damage the thyroid. Therefore, iodine-loaded hydrogels not only inhibited OS *in vivo* growth but also exhibited good biosafety.

In this study, we constructed self-assembled imageable silk hydrogels as a controlled release system for the local release of iodine for OS treatment, a novel attempt to avoid multiorgan damage caused by conventional chemotherapeutic drugs and major surgical trauma. Self-assembling imageable silk hydrogels could be injected *via* syringe, which mad transdermal injection to the interior of the OS possible, as the injection was far less invasive than surgery. MD allowed the hydrogel to be imageable with X-rays; thus, the hydrogel could be injected inside the OS with X-ray guidance, which was beneficial for injecting hydrogels and delivering anti-OS drugs to local lesions. Moreover, the systemic damage caused by iodine was far less severe than that caused by chemotherapeutic drugs. No abnormalities were found in any of the seven types of organs we collected, and the iodine-loaded hydrogel achieved excellent results in the treatment of OS, which few chemotherapeutic drugs can achieve. Of course, hydrogels also have some limitations such as low mechanical strength and limited support, which might increase their risk of fracture. To address the limitations of hydrogels that may be encountered in future applications, it is possible to combine the application of hydrogels with external fixation and use the supportive properties of external fixation to compensate for the lack of mechanical strength and support of hydrogels. With regard to the risk of metastasis, we were also concerned at the beginning of the study that injecting a volume of hydrogel solution into an OS might lead to an increase in local pressure, thus increasing the risk of OS metastasis. However, bioluminescence was not observed at sites other than OS by *in vivo* bioluminescence imaging at the end of the *in vivo* trial. No cells with tumour cell characteristics were observed in the organs. In this study, hydrogel injections were not found to increase the risk of OS metastasis.

We conducted only a short-term assessment of systemic injury; we will evaluate the long-term systemic damage, therapeutic effect and metastasis in the *in situ* OS model in subsequent experimental studies. There are various death receptors, such as Fas, tumour necrosis factor receptor 1, tumour necrosis factor-related apoptosis-inducing ligand receptor 1, tumour necrosis factor-related apoptosis-inducing ligand receptor 2, activation of death receptor-4 and activation of death receptor-5. Fas/FasL is the classic receptor and ligand of the death receptor pathway, and although some studies have reported the role of Fas in osteosarcoma, there are few studies on the tumour-inhibiting effect of iodine. Therefore, we chose the receptor-ligand pair Fas and FasL to investigate whether iodine can induce apoptosis in osteosarcoma cells by activating the death receptor pathway. In our subsequent studies, we will examine other receptors and ligands in the death receptor family. In addition, no chemotherapeutic drugs were used in this study, which reduced the emission of pollutants. During the experiment, all invasive operations were carried out under anaesthesia. The experimental animals felt minor pain or no pain during and after the operation. With these measures, the expected experimental results were obtained, and the welfare of the experimental animals was ensured.

## Conclusion

We demonstrated that iodine induced apoptosis in MG-63 and Saos-2 cells by regulating the death receptor pathway. Imageable hydrogels could be appropriately injected and localized with X-ray imaging. The operation was simple and minimally invasive. Moreover, iodine-loaded hydrogels were safe, had good biosafety and could control the release of iodine, which minimized systemic side effects and maximized the therapeutic impact. Our results suggested that self-assembling imageable silk hydrogels had good biosafety, were easy to operate, were minimally invasive and had substantial anti-OS effects. Our study promises efficient, systemic and locally less invasive treatment of OS in the future, which might replace the current treatment strategies used for OS.

## Data Availability

The raw data supporting the conclusion of this article will be made available by the authors, without undue reservation.
