# Supplementary material for: Self-Assembling Imageable Silk Hydrogels for the Focal Treatment of Osteosarcoma
Source: Front Cell Dev Biol. 2022 Jun 20;10:698282. doi: 10.3389/fcell.2022.698282 (PMC9251127; doi:10.3389/fcell.2022.698282)
Supplement: Supplementary file 1 [file Table1.pdf]

Supplementary materials

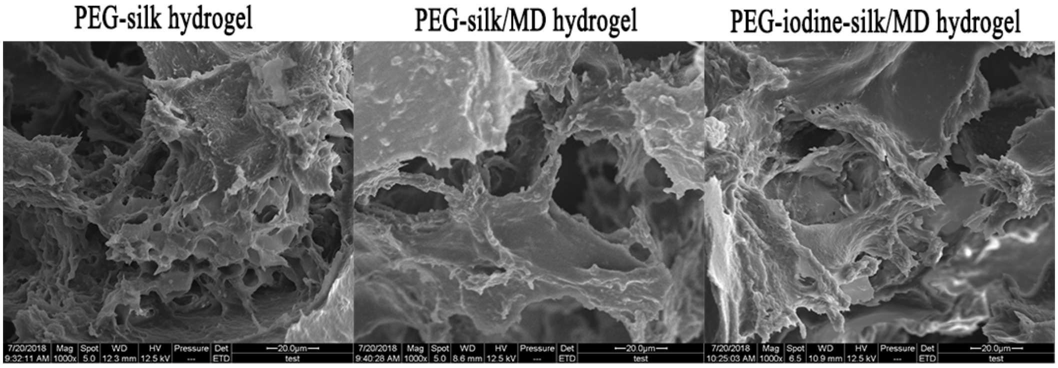

Supplement Figure 1. Silk hydrogel morphologies determined by SEM. The internal network of the gel is small and compact, and we can see the silk fibroin, nanospheres, nanofilaments and microfibres formed during gelation (scale bars = 20 μm) (SEM: scanning electron microscopy, PVP-I: polyvinylpyrrolidone iodine, PEG: polyethylene glycol, MD: meglumine diatrizoate)

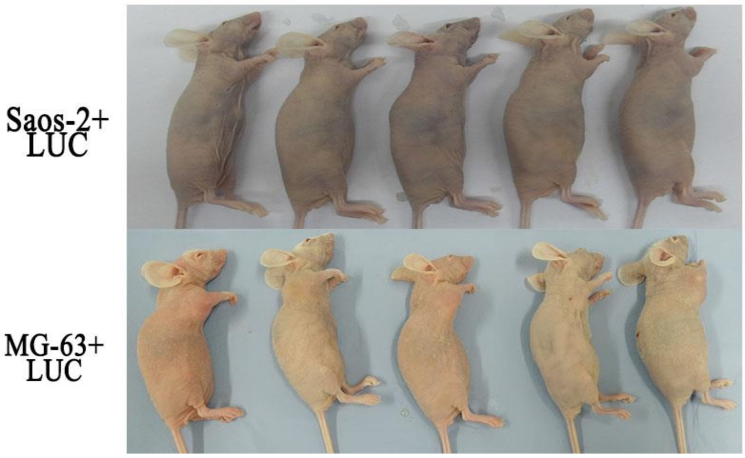

Supplement Figure 2. Saos-2+LUC and MG-63+LUC cells tumour formation analysis by macroscopic. No tumour was seen in the right axilla of the 5 Saos-2+LUC injection nude mice. MG-63+LUC cells formatted tumours on the right axilla of the 5 nude mice.
